# The significance of macrophage polarization subtypes for animal models of tissue fibrosis and human fibrotic diseases

**DOI:** 10.1186/s40169-015-0047-4

**Published:** 2015-02-07

**Authors:** Peter J Wermuth, Sergio A Jimenez

**Affiliations:** Jefferson Institute of Molecular Medicine, Thomas Jefferson University, Bluemle Life Science Building Suite 509, 233 South 10th Street, Philadelphia, PA 19107-5541 USA

**Keywords:** Monocytes, Macrophages, Macrophage polarization, Fibrosis, Chemokines, Cytokines, Fibrosing disorders

## Abstract

The systemic and organ-specific human fibrotic disorders collectively represent one of the most serious health problems world-wide causing a large proportion of the total world population mortality. The molecular pathways involved in their pathogenesis are complex and despite intensive investigations have not been fully elucidated. Whereas chronic inflammatory cell infiltration is universally present in fibrotic lesions, the central role of monocytes and macrophages as regulators of inflammation and fibrosis has only recently become apparent. However, the precise mechanisms involved in the contribution of monocytes/macrophages to the initiation, establishment, or progression of the fibrotic process remain largely unknown. Several monocyte and macrophage subpopulations have been identified, with certain phenotypes promoting inflammation whereas others display profibrotic effects. Given the unmet need for effective treatments for fibroproliferative diseases and the crucial regulatory role of monocyte/macrophage subpopulations in fibrogenesis, the development of therapeutic strategies that target specific monocyte/macrophage subpopulations has become increasingly attractive. We will provide here an overview of the current understanding of the role of monocyte/macrophage phenotype subpopulations in animal models of tissue fibrosis and in various systemic and organ-specific human fibrotic diseases. Furthermore, we will discuss recent approaches to the design of effective anti-fibrotic therapeutic interventions by targeting the phenotypic differences identified between the various monocyte and macrophage subpopulations.

## Introduction

Monocytes/macrophages are a heterogeneous cell population that plays a crucial role in various fundamental innate immunity processes displaying remarkable flexibility for adaptation to specific external and internal stimuli [1,2]. The monocyte/macrophage system consists of: 1) bone marrow-derived circulating monocytes recruited to sites of tissue injury; 2) infiltrating macrophages arising from *in situ* differentiation of recruited monocytes; 3) resident tissue macrophages derived from embryonic yolk-sac precursors such as hepatic Kupffer cells, pulmonary alveolar and interstitial macrophages, renal intraglomerular mesangial cells, and dermal Langerhans cells, and 4) monocyte-derived dendritic cells that present antigen in the context of MHC molecules, representing an important bridge between the innate and adaptive immune systems. Monocytes/macrophages play important roles in wound healing and in the response to tissue injury. Properly orchestrated wound repair accomplishes the elimination of injurious initiating factors and the restoration of the damaged tissue architecture and integrity. However, dysregulation of this process can result in pathologic fibrosis. Further, there is abundant experimental evidence that monocytes/macrophages and the molecular pathways activated during wound healing also play a role in the pathogenesis of systemic fibroproliferative diseases such as Systemic Sclerosis (SSc), and Nephrogenic Systemic Fibrosis (NSF), as well as organ-specific fibrotic disorders including Idiopathic Pulmonary Fibrosis (IPF) and liver, kidney, and cardiac fibrosis.

### Origin and diversity of monocytic phagocytes

**Figure 1:**
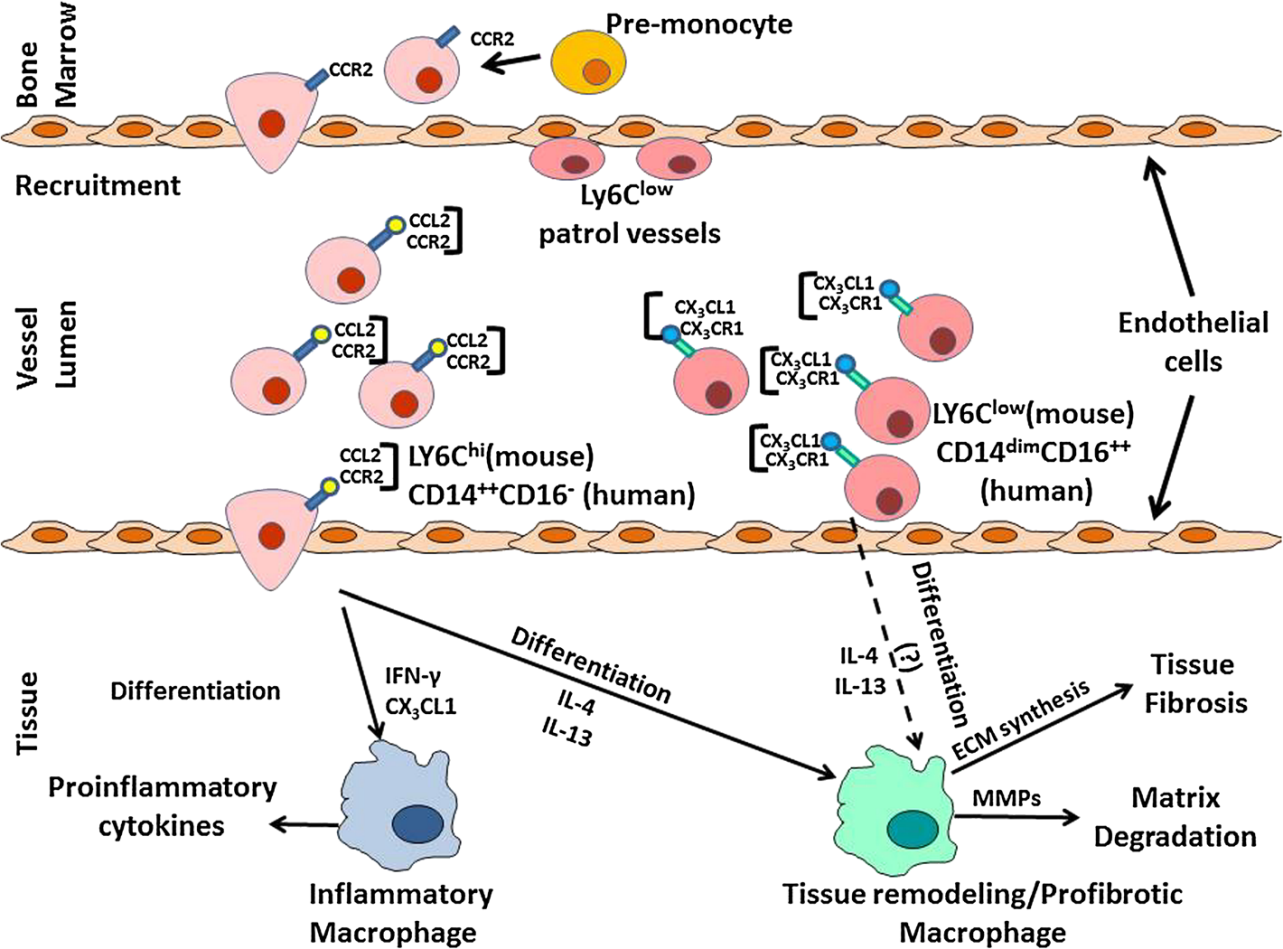
**The monocyte/phagocyte system.** Two populations of bone-marrow derived monocytes, Ly6C^hi^ and Ly6C^lo^ monocytes enter the circulation. Ly6C^lo^ monocytes are primarily responsible for patrolling the vasculature, regulating neovascularization and monitoring endothelial cell homeostasis. Ly6^hi^ monocytes enter the tissues as part of their intrinsic function or in response to pro-inflammatory chemokines released at sites of injury by resident tissue macrophages. Upon entering the tissue, these monocytes can differentiate into macrophages in response to various inflammatory and immune stimuli specific to the tissue microenvironment. The phenotype of the differentiated infiltrating macrophages can be broadly classified as classically activated inflammatory (M1) or alternatively activated tissue remodeling/profibrotic (M2) macrophage populations. Resident tissue macrophages that originated from embryonic yolk sac and migrated to organs during development can be replenished either by proliferation of the resident population or by infiltrating differentiated macrophages that have survived following inflammation resolution. Solid arrows indicate major differentiation/activation pathways. Dotted arrows indicate secondary or minor differentiation pathways. **CCL1**, C-C chemokine ligand 1; **CCR1**, C-C chemokine receptor 1; **CCR2**, C-C chemokine receptor 2; **CCR5**, C-C chemokine receptor 5; **CX**_**3**_ **CL1**, C-X_3_-C chemokine ligand 1; **CX**_**3**_ **CR1**, C-X_3_-C chemokine receptor 1; **GC** glucocorticoid; **IFN-γ**, Interferon-γ; **IL-4**, Interleukin-4; **IL-10**, Interleukin-10; **IL-13**, Interleukin-13; **TGF-β**, Transforming Growth Factor β.

Bone marrow-derived monocytes are recruited into tissues from the peripheral blood, differentiating *in situ* and joining the pool of resident tissue macrophages to mount a defense against pathogens and assist in wound healing. Monocyte/macrophage differentiation is regulated by a complex interplay of factors influenced by the local tissue environment and by features of the specific injury initiator as illustrated in Figure 1 [3-6]. Mirroring the Th1/Th2 T-helper cell nomenclature, monocytes and macrophages have traditionally been divided into two major subpopulations based on their origin, location and their *in vitro* response to cytokines or microbial products as summarized in Table 1. In this scheme, monocytes/macrophages that mediate inflammation have been classified as M1 whereas monocytes/macrophages with tissue remodeling/profibrotic activity have been classified as M2. Although this classification scheme is now generally considered to be a gross oversimplification of the true spectrum of *in vivo* monocyte/macrophage phenotypes, numerous studies still attribute an M1-like or M2-like phenotype to specific subpopulations of mouse or human monocytes/macrophages. For the purpose of this review, we will refer to these populations as either inflammatory or tissue remodeling/profibrotic, respectively, however, we will indicate whether the authors used the M1 or M2 classification in parentheses.

**Table 1:** **Main monocyte and macrophage polarization population subsets**

	**Circulating monocytes**		**Differentiated macrophages**			
**Phenotype**	**Inflammatory**	**Anti-inflammatory**	**Inflammatory**	**Tissue remodeling/profibrotic**		
**Classical nomenclature**	**Ly6C** ^**hi**^ **(mouse)**	**Ly6C** ^**low**^ **(mouse)**	**M1**	**M2a**	**M2b**	**M2c**
	**CD14** ^**++**^ **CD16** ^**-**^ **(human)**	**CD14** ^**dim**^ **CD16++ (human)**				
**Differentiating agent**			IFNγ	IL-4	ICs	IL-10
			TNF-α	IL-13	LPS	TGF-β
			LPS	IL-4 + LPS	LTR + IL-1R	GC
						IFN-β
						HDL
**Markers**	CCR1^hi^	CCR2^low^	CD86	CD163	CD86	CD163
	CCR2^hi^	CCR5^hi^	CD80	MHCII	MHCII	TLR1
	CX_3_CR1^low^	CX_3_CR1^hi^	MHCII^hi^	SR		TLR8
	CD11b^+^	CD11b^+^	IL-1R	CD206		
	CD115^+^	CD115^+^	TLR2	TGM2		
	CD62L^+^	CD62L^-^	TLR4	DecoyR		
	CD11c^-^	CD11c^+^	INOS			
**Cytokines produced**	TNF-α	IL-10	TNF-α	IL-10	IL-4	IL-10
	IL-1β	TGF-β	IL-1β	TGF-β	IL-6	TGF-β
			IL-6	IL-1ra	IL-10	
			IL-12		TNF-α	
			IL-23			
**Chemokines produced**	CCL2		CCL8-11	CCL17	CCL1	CCR2
			CCL2-5	CCL22		
				CCL24		

Classification scheme for human and murine monocyte and macrophage population subsets displaying their response to differentiating agents, expression of cell surface markers and the production of major cytokines and chemokines.

Functional monocyte/macrophage classification based on *in vitro* modes of activation is widely employed, however, currently there is no universally accepted nomenclature. Recently, two proposals have been made to revise the monocyte/macrophage system nomenclature to more accurately represent the expanded phenotype diversity within this system and to incorporate the rapidly expanding database of surface marker expression and transcriptomic analyses delineating steady-state monocyte/macrophage populations in humans and mice. One such proposal is based on macrophage origin, differentiation activators, and macrophage activation marker expression [7]; and the other is based on their cellular ontogeny [8].

### Circulating monocytes

Murine circulating monocytes have been classified into two populations based on the relative expression of the Ly6C surface molecule, Ly6C^hi^ and Ly6C^low^, whereas in humans three monocyte populations have been identified based on the relative expression of CD14 and CD16. These subpopulations are identified as CD14^++^CD16^-^, CD14^++^CD16^+^, and CD14^+^CD16^++^ [9-11]. Murine classical (Ly6C^hi^) or “inflammatory monocytes” [11-13] respond to inflammatory signals, such as IFN-γ, TNF-α, or lipopolysaccharide (LPS) and leave the circulation by extravasation, whereas non-classical (Ly6C^low^) “anti-inflammatory” or “wound healing” monocytes influence neovascularization and monitor endothelial cell homeostasis by patrolling the luminal side of the vasculature [14,15]. Recently, Ly6C^hi^ monocytes that constitutively traffic into skin, lungs, and lymph nodes during homeostasis but do not differentiate into macrophages or dendritic cells in the tissues have been identified. The transcriptome of these cells is similar to that of circulating monocytes except for a limited number of genes including MHCII and Cox-2. Although these cells can bind and shuttle antigen to the lymph nodes, they are incapable of blood recirculation [16]. In the corresponding human subpopulations, the “classical” pro-inflammatory CD14^++^CD16^-^ subset expresses CCR2, CD62L (L-Selectin) and FcγRI (CD64) whereas “non-classical” anti-inflammatory CD14^dim^CD16^++^ monocytes lack CCR2 and display higher levels of FCγRII (CD32) and MHCII [11,13,15].

An area of debate concerns whether the Ly6C^hi^ and Ly6C^low^ represent separate cell populations or whether Ly6C^low^ monocytes arise only after differentiation from Ly6C^hi^ monocytes followed by return to the bone marrow from the circulation. Several recent studies indicate that only Ly6C^hi^ monocytes leave the circulation by extravasation in response to inflammatory signals, whereas Ly6C^low^ monocytes reside within the vasculature [14,15]. In most injured tissues, the first infiltrating cells are Ly6C^hi^ monocytes followed by a decline in their numbers with a concomitant increase of Ly6C^low^ monocytes [17-24]. In mouse myocardium [17], kidney [18], muscle [21], and lung [23,24], the Ly6C^low^ population arises from *in situ* differentiation of the recruited Ly6C^hi^ population whereas, in wounded mouse skin, a sequential invasion of Ly6C^hi^ monocytes followed by Ly6C^low^ monocytes is observed [20,22].

### Infiltrating macrophages

Macrophage populations are generally classified based on the differential expression of cell surface markers, phenotypic differences in their response to stimuli, or their origin and location [7,8,25-34]. The complex process of monocyte recruitment and their subsequent differentiation into various macrophage subpopulations is depicted in Figure 1. Exposure of peripheral Ly6C^hi^ monocytes to GM-CSF polarizes them to differentiate into macrophages with inflammatory properties whereas CSF-1 exposure of peripheral Ly6C^hi^ monocytes induces Ly6C downregulation followed by polarization to tissue remodeling/profibrotic (M2) macrophages [9-11,25]. M1 or “classically activated” macrophages are induced following recruitment by either IFN-γ alone or in conjunction with the microbial product LPS, or by cytokines such as TNF-α or GM-CSF. Classically activated inflammatory macrophages present antigen, express CD80, CD86, IL-1R, TLR2, TLR4 and iNOS, and possess a proinflammatory phenotype characterized by the production of the cytokines TNF-α, IL-1β, IL-6, IL-12, IL-23 and of the chemokines CCL2-CCL5, CCL8-CCL11. Alternatively activated tissue remodeling/profibrotic macrophages were initially defined as being activated by exposure to IL-4 or IL-13, however, other stimuli can induce polarization to alternatively activated macrophages, resulting in subdivision of tissue remodeling/profibrotic macrophages into three subtypes. One subtype is activated by exposure to IL-4 or IL-13; the second subtype is induced by IL-10, TGF-β, glucocorticoids or IFN-β; and the third subtype is induced by exposure to immune complexes. Caution must be used, however, in translating phenotypes of monocytes/macrophages differentiated *in vitro* to *in vivo* models since factors such as cell maturation, variations in extracellular matrix (ECM) composition within a specific tissue, and chemoattractants not precisely replicated in the *in vitro* differentiation protocols can produce alterations in the functional properties of these cells not observed *in vitro*. The recent characterization of mouse and human monocyte/macrophage subpopulation transcriptomes and gene networks should allow a more accurate translation of *in vitro* and *in vivo* animal model data to human diseases [35-41].

Recent studies have shown that besides changes in transcriptome expression modifications in non-coding RNA species and changes in epigenetic events may also be involved. Indeed, a recent examination demonstrated a role of miRNA on the differentiation of recruited monocytes into macrophages by measuring the response of human peripheral blood mononuclear cells (PBMC) to various differentiation stimuli *ex vivo* [42]. It was found that the monocyte miRNA profile varied in a stimulus and time-dependent manner. Exposure of PBMC to LPS, an inducer of inflammatory monocyte/macrophage (M1) differentiation caused increased miR-193b and miR222 and decreased miR27a, whereas exposure to IFN-γ, also considered an inflammatory monocyte/macrophage (M1) inducer, caused decreased miR-222, miR-125a-5p and miR-27a. IL-4, an inducer of tissue remodeling/profibrotic monocyte/macrophage (M2) differentiation increased miR-193b and miR-222 but decreased miR125-5p. The kinetics of the changes in the individual miRNA levels also varied. Of particular interest was that co-culture of PBMC with CD14^-^ non-monocytic cells from the same donor induced accumulation of miR-155 and miR193b and increased expression of NF-κB, STAT1 and of the interferon-responsive genes ITGB7 and ISG20. The NF-κB repressor IκBα decreased in co-cultured monocytes, providing the mechanistic basis for NF-κB activation. These results collectively indicate that microenvironmental factors can uniquely modify the phenotype of differentiated recruited monocytes inducing marked changes in mRNA levels and also in miRNA expression and kinetics that may modulate the remarkable flexibility of monocyte responses to specific environmental cues.

### Resident tissue macrophages

Resident tissue macrophages arising from embryonic yolk-sac precursors display tissue-specific phenotypes determined by microenvironmental factors and often cannot be classified into the inflammatory (M1) or tissue remodeling/profibrotic (M2) categories [43-47]. Although it is generally accepted that resident tissue macrophages have limited self-renewal capacity and are replenished from the pool of circulating monocytes, recent studies demonstrate that resident macrophages in certain tissues are replenished primarily through their own proliferation [47-49], although circulating blood monocytes can repopulate cardiac resident macrophages [50].

Since a specific macrophage phenotype is not irreversibly encoded in their DNA, monocytes and macrophages maintain the flexibility necessary to adapt to tissue changing conditions and this flexibility is an important component of their first-response role in tissue injury. Furthermore, the *in vivo* monocyte/macrophage phenotype differentiation process most likely represents a fluctuating spectrum of polarization states that is constantly and reversibly modified in response to changing microenvironmental conditions [51,52], and that the previously characterized monocyte/macrophage subsets do not describe fixed subpopulations. More extensive analyses of monocyte/macrophage transcriptomes, aimed at the identification of common and unique activation pathways, will likely reveal the relevance of these transcriptional changes to important physiological processes and to the pathogenesis of diseases in which monocytes/macrophages play a role.

### Dendritic cells

Dendritic cells (DCs) are motile cells with stellate morphology originated from bone marrow derived circulating monocyte precursors. Dendritic cells express high levels of MHC class II and the integrin CD11c, migrate from tissues to secondary lymphoid tissues, and can capture, process and present antigen [53,54]. Immature DCs possess high phagocytic activity that triggers a complex maturation process characterized by upregulation of various surface molecules including class II MHC, CD80, CD83, CD86 and CD40 that enable antigen presentation and interaction with T cells. Two major subpopulations of DCs have been described: plasmacytoid DCs (pDCs) and classical or myeloid DCs (cDCs) [55]. pDCs are a long-lived population present in the bone marrow and all peripheral organs characterized by massive production of type I interferons in response to viral infections, enabled by constitutive expression of IFN regulatory factor 7 (IRF7). pDC also have the ability to act as antigen-presenting cells capable of initiation and regulation of T cell responses [56]. Human pDCs display a CD4^+^ CD303^+^ CD68^+^ phenotype whereas murine pDCs express CD11c^low^ B200^+^ Ly6C^+^ CD45RA^+^ phenotype as well as CD217 (BST-2) and SiglecH [57,58]. pDCs express only TLR7 and TLR9, allowing viral single-stranded RNA and unmethylated DNA to activate TLR pathway proinflammatory signaling [59].

cDCs refer to all DCs other than pDCs and comprise short-lived highly migratory cells that populate most lymphoid and nonlymphoid tissues that migrate to the secondary lymphoid organs by afferent lymphatics following phagocytosis and regulate T cell activity by mediating the adaptive immune response to foreign antigen and by maintaining tolerance to endogenous antigen in both the steady state tissue and during infection [60,61]. The major cDC population displays a CD11c^+^ phenotype in humans whereas in mice the major cDC population expresses a CD11c^+^ CD11b^-^ CD45RA^-^. Both cDC subpopulations express TLR4 and TLR9 [60]. In both mice and humans, a minor cDC population displays a CD141^+^ CLEC9A^+^ phenotype and is a major producer of IFN-β. These cells are capable of presenting antigen to C8^+^ cytotoxic T cells [62,63]. TLR signaling in either pDCs or cDCs results in production and secretion of IL-12, IL-6, TNF-α, and the chemokines CCL3, CCL4, CCL5, CXCL9 and CXCL10 besides type I IFNs [64].

### Role of Monocytes/macrophages in wound repair

**Figure 2:**
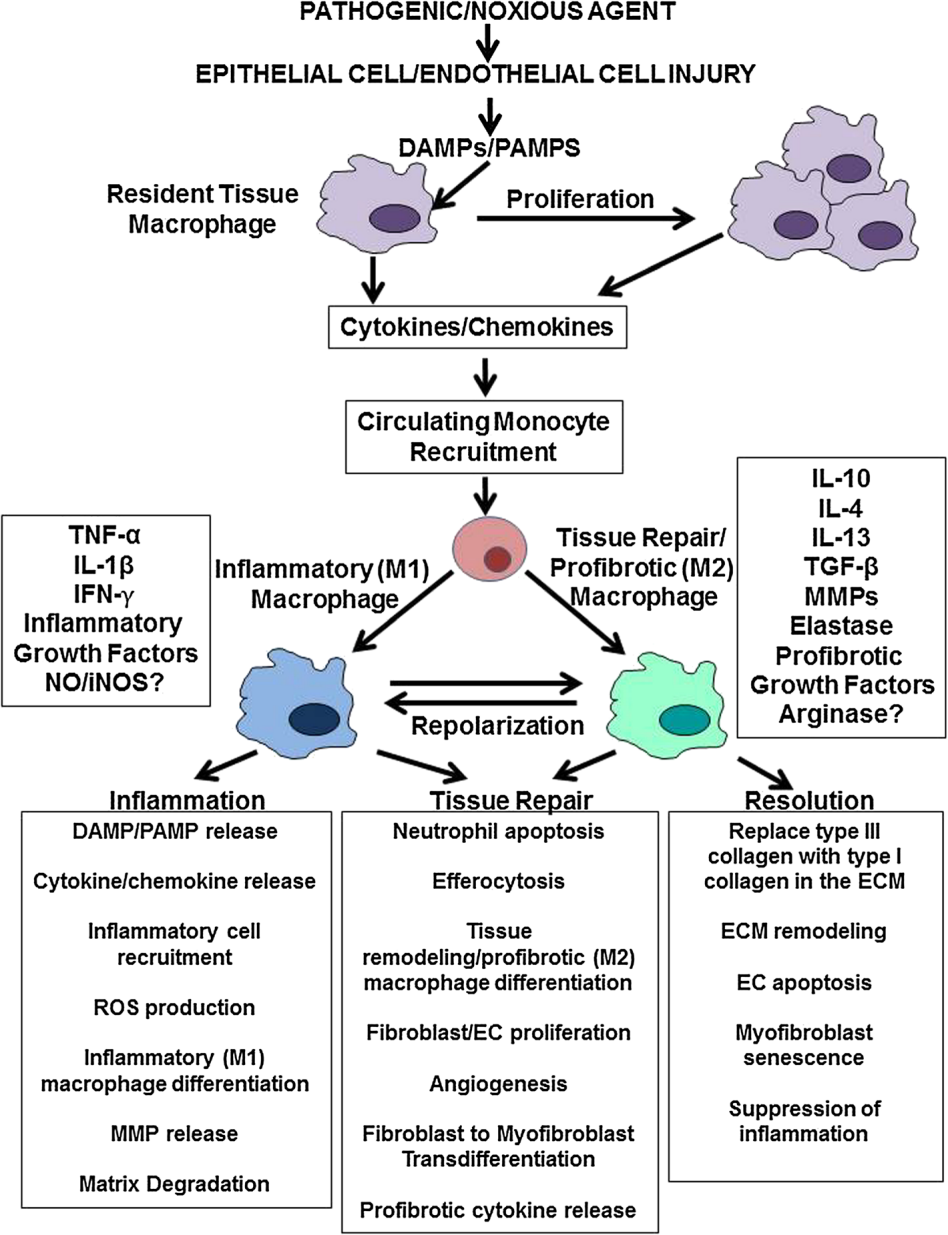
**Role of monocytes/macrophages in wound healing.** Injury to epithelial and/or endothelial cells caused by various exogenous or endogenous factors resulting in tissue damage triggers complex interconnected wound-healing programs to restore tissue homeostasis. Release of DAMPs and PAMPS by the damaged tissue triggers an inflammatory response that activates resident tissue macrophages, stimulating their proliferation and initiating the recruitment of inflammatory Ly6C^hi^ monocytes and neutrophils to the wound. Cytokines and chemokines produced and secreted by local epithelial, endothelial and innate immune cells subsequently influence the differentiation and polarization of the recruited monocytes into inflammatory classically activated M1 macrophages. The M1 macrophages also stimulate the transdifferentiation of resident quiescent fibroblasts into activated myofibroblasts that synthesize a provisional matrix as well as stimulate endothelial cell and fibroblast proliferation and orchestrate angiogenesis. Recruited monocytes respond to the different cytokine profiles, differentiating into M2 macrophages and initiating tissue repair with upregulated MMP secretion followed by suppression of any remaining inflammation and synthesis of a permanent extracellular matrix by activated myofibroblasts. **DAMPs**, Damage associated molecular patterns; **EC**, Endothelial cell; **ECM**, Extracellular matrix; **IFN**, Interferon; **iNOS**, Inducible nitric oxide synthase; **IL**, Interleukin; **MMP**, Matrix metalloproteinase; **PAMPs**, Pathogen associated molecular patterns; **TGF-β**, Transforming growth factor, beta.

The wound healing process is mediated by various intracellular, autocrine, and paracrine pathways initiated following pathogen infection or exposure to chemical toxins or other stimuli that induce tissue damage [65-74]. This process requires a coordinated response from various cell types, including endothelial cells, keratinocytes, fibroblasts and various immune cells. The molecular pathways triggered in these cells induce marked changes in gene expression causing modulation of their cellular phenotypes resulting in proliferation, differentiation or migration. The wound healing process restores tissue integrity and repairs the damage induced by the initial injury and by the associated inflammatory response. As illustrated in Figure 2, this process consists of three major overlapping stages: 1) an inflammatory stage in which immune cell recruitment to the injury site induces production of proinflammatory cytokines, chemokines and reactive oxygen species (ROS) eliminating invading pathogens and removing necrotic tissue and cellular debris; 2) a proliferative stage in which proliferation and activation of fibroblasts, keratinocytes and endothelial cells mediates the production of a provisional ECM, re-epithelialization, and neoangiogenesis to restore normal blood supply to the injured area; and 3) a resolution stage characterized by suppression of inflammation and apoptosis of recruited cells followed by wound remodeling and provisional matrix replacement with a more durable ECM capable of providing tensile strength to the newly formed tissue.

### The inflammatory stage

The inflammatory stage is triggered by an initiating pathogen or toxin that results in the release of pathogen- or damage-associated molecular patterns (PAMPs or DAMPs, respectively) that ligate and activate pattern recognition receptors such as toll-like receptors (TLRs), NOD-like receptors (NLRs), C-type lectin receptors (CLRs) or other receptors on resident cells, including endothelial cells, mast cells, tissue macrophages and interstitial fibroblasts [75,76]. Receptor activation triggers the production and secretion of cytokines, chemokines and growth factors that induce inflammation and the recruitment of inflammatory cells, primarily neutrophils and monocytes [77]. The recruited monocytes are proinflammatory and will subsequently differentiate into inflammatory (M1) macrophages. The activated resident cells and the recruited inflammatory (M1) macrophages release toxic ROS that destroy invading pathogens [78,79] and induce the expression of genes encoding various cytokines, inflammatory molecules and multiple proteases including MMPs, serine and cysteine proteases, and elastases.

Removal of damaged cells and degradation of the ECM releases matrix-bound growth factors such as TGF-β and cytokines that stimulate endothelial cell and fibroblast proliferation, preparing the tissue for neo-vascularization and tissue repair [80,81]. ROS production, however, also damages endogenous cells and one of the most important functions of the recruited monocytes besides removal of pathogens is the removal of necrotic resident cell debris. Macrophages also induce neutrophil apoptosis and phagocytose the remains [82-84]. This represents an important trigger for inflammation resolution and the initiation of tissue repair [85-91]. Several mechanisms are involved in these effects including alterations of the balance between proinflammatory and anti-inflammatory cytokine secretion [87-91], downregulation of proinflammatory transcription factors important for neutrophil survival such as NF-κB and IRF1 [92,93], and concomitant upregulation of the anti-inflammatory transcription factor IRF-4 [94,95]. These effects collectively promote inflammatory (M1) to wound healing/profibrotic (M2) macrophage phenotype transition and initiate the resolution of inflammation [86-91].

### Tissue repair/wound healing phase

The transition from an inflammatory phenotype (M1) to a wound healing/profibrotic (M2) phenotype induces the progression from the inflammation phase to the tissue repair phase (Figure 2). Monocytes/macrophages at the transition between the inflammatory and tissue repair/wound healing stages produce copious amounts of cytokines and growth factors that promote the proliferation of multiple cell types involved in damaged tissue repair [96-100]. Wound healing/profibrotic (M2) macrophages appear at this stage, either via differentiation of newly recruited infiltrating monocytes or by *in situ* transition of previously differentiated infiltrating inflammatory (M1) macrophages to a wound healing/profibrotic (M2) phenotype. STAT6 is activated during this transition and promotes IL-4/IL-13-mediated differentiation of wound healing/profibrotic (M2) macrophages by upregulating their expression of arginase (Arg1) and multiple other wound healing/profibrotic phenotype genes [101]. Wound healing/profibrotic macrophages possess an anti-inflammatory phenotype and stimulate and activate fibroblasts towards their increased ECM production and secretion [96-101]. The macrophage phenotype is also influenced by changes in the mechanical, cellular and metabolic characteristics of the target tissue [102-104].

The wound healing/profibrotic macrophages in the late tissue repair stage initiate the transition to the resolution/tissue remodeling stage. They also support endothelial cell survival and release TGF-β that triggers transdifferentiation of fibroblasts to activated myofibroblasts [72,73], a unique population of mesenchymal cells that display increased production of fibrillar type I and type III collagens, expression of α-smooth muscle actin (α-SMA) and a reduction in the expression of ECM degradative enzymes such as the MMPs [105-114]. More recent studies have demonstrated that endothelial cells may also undergo a phenotypic transition into profibrotic mesenchymal cells, a process known as endothelial to mesenchymal transition (EndoMT), following stimulation by TGF-β produced by the macrophages present in the tissue [115-124].

### Resolution/remodeling stage

In the final phase, the wound scar tissue is remodeled by replacement of the provisional ECM with a stronger, durable ECM, characterized by extensive collagen cross-linking and the gradual replacement of type III collagen with type I collagen [125-127]. These changes are followed by senescence or apoptosis of activated myofibroblasts [128,129] and regression of the neovasculature [130-132]. The macrophage phenotype during this stage remains unclear with some studies in the liver suggesting the existence of a unique population of fibrinolytic scar-associated macrophages required for fibrosis resolution [86,133-136], although others have reported these macrophages to be profibrotic [137,138].

### Monocytes/macrophages in tissue fibrosis

Dysregulation of normal wound healing results in pathologic fibrosis and the replacement of normal tissues with fibrotic tissue causing progressive loss of function in the affected organs [139-142]. Chronic fibroproliferative diseases account for a large proportion of total worldwide mortality [143-146] and the lack of effective fibrotic disorder treatment represents a substantial unmet need. Fibrosis can result from the activation of various common pathways as well as from activation of tissue-specific pathways with unique consequences for fibrosis progression in the particular organ. Various cells types including endothelial cells, epithelial cells, activated myofibroblasts and macrophages participate in one or more steps in the fibrotic process [147-149]. Most fibrotic diseases also manifest substantial vascular remodeling with impaired angiogenesis followed by progressive obliteration of blood vessels that is often an early feature suggesting that endothelial injury may be a precipitating event in fibrosis pathogenesis [150-152].

It is important to note in any discussion of research on the role of monocytes and macrophages in the initiation and persistence of tissue fibrosis in human disease that the majority of investigations employ animal models, most commonly mice to identify the molecular pathways important in the regulation of this process. Studies of monocyte/macrophage involvement in patients with these diseases tend to be primarily descriptive, examining the macrophages present in samples from patients with the disease compared to normal controls. Each approach has its drawbacks and it is essential to consider these drawbacks when attempting to draw conclusions from comparisons of experimental results from one model system to the other. Studies conducted with human samples offer little opportunity for experimental manipulation of differentiation conditions and often are reflective of only a single timepoint during the course of a highly dynamic process. Also, because even small changes in tissue microenvironment can induce major changes in monocyte/macrophage phenotypes, the relevance of results from the experimental manipulations performed in animal models to human disease may be limited. Therefore, the importance of characterizing the monocyte/macrophage populations involved employing multiple methodologies and functional characteristics cannot be underestimated, particularly for the indentification of specific populations to be targeted to modulate the fibrotic process. Recently, with the advent of systematic studies exploiting advances in transcriptome analysis and the identification of expression of multiple cell surface markers from large sample sets of human and murine immune cells from the Human Immunology Project Consortium and the Immunological Genome Project, respectively, a valuable and growing resource is being generated that should allow for more consistent and accurate translation of experimental results obtained from animal models of tissue fibrosis to their human disease counterparts [35-41]. Another valuable resource that may allow for better evaluation of the role of molecular pathways identified using animal models or *in vitro* analysis of human monocytes/macrophages is the generation of the MITRG and MISTRG mouse strains carrying human versions of cytokine encoding genes important in human innate immune cell development [153]. Injection of human fetal liver or adult CD34^+^ progenitor cells into these mice results in the humanized development and function of monocytes, macrophages and NK cells, generating a model that may allow the evaluation of therapeutic candidates for monocyte/macrophage-mediated tissue fibrosis in an *in vivo* milieu more reflective of the human physiology.

Monocytes and macrophages play a pivotal role in coordinating the various stages of the wound healing process by their ability to produce and secrete numerous growth factors, cytokines and other mediators capable of exerting paracrine effects on other cells in affected tissues [27,59-72,154-157]. Failure of the early inflammatory phase of the wound healing process to resolve, as indicated by the persistence of inflammatory macrophages, causes sustained and excessive synthesis and deposition of ECM components, leading to fibrotic tissue formation [158,159], and their shift into the wound healing/profibrotic macrophage phenotype further increases tissue fibrosis, through their increased and persistent expression of TGF-β [160] and through their induction of a change in the balance of ECM metabolism toward ECM deposition and accumulation [161-168]. However, it should be emphasized that an anti-fibrotic function for wound healing/profibrotic macrophages has been demonstrated in both human and mouse models [169-172]. Furthermore, other investigations have indicated either a primary or secondary role for inflammatory (M1) macrophages with CCR8 [173], CCR4 [174] and Annexin A1 [175] implicated as important regulators of this process. However, it has been demonstrated that both inflammatory (M1) and wound healing/profibrotic (M2) populations participate in fibrosis and suggest a requirement for inflammation or defective inflammation resolution in fibrosis pathogenesis [176-179].

### Role of TLR-macrophage interactions in tissue fibrosis

The role of TLR signaling in fibrosis-associated inflammation and macrophage activation is an area of intense interest. TLR signaling mediates certain pathways in fibrotic diseases such as SSc and NSF [180-186]. Expression of TLR4 and its coreceptors MD2 and CD14 is increased in human lesional SSc skin and this increase correlates with disease severity and progression [187]. Chronic dermal LPS exposure in mice induces expression of proinflammatory chemokines and upregulation of various TGF-β regulated genes as well as recruitment and activation of both inflammatory (M1) and tissue remodeling/profibrotic (M2) macrophages [187]. TLR2, TLR4 and MyD88 expression is increased in mice with renal fibrosis induced by unilateral ureteral obstruction (UUO) with concomitant increases in tissue remodeling/profibrotic (M2) macrophage infiltration and collagen deposition [188]. Tissue remodeling/profibrotic (M2) macrophage depletion but not T-cell depletion abrogated UUO-mediated fibrosis. Signaling through these receptors triggered a TH_2_ immune response with increased expression of IL4 followed by IL-10 and TGF-β expression, increased macrophage infiltration and exaggerated collagen deposition. All these changes were abrogated in TLR2, TLR4 and MyD88 knockout mice indicating the crucial role of TLR and MyD88 in their occurrence. In contrast to these results, Staphylococcus aureus biofilm infection of MyD88 knockout mice resulted in the increased recruitment of activated M2 macrophages and fibrosis development in a TLR-independent manner, possibly involving IL-1R, IL-18R or IL-33R activity [189]. It has also been shown that during ischemia/reperfusion-induced kidney injury in mice, various DAMPs are generated including the calcium-binding protein S100A8/A9 complex that activates TLR4 signaling. However, S100A8/A9 knockout mice display enhanced renal damage and fibrosis that correlates with increased expression of M2 macrophage markers raising some controversy about the role of S100A8/A9 in TLR mediated fibrosis [190].

### Monocyte/macrophage polarization phenotype in human fibrotic diseases and animal models of tissue fibrosis

There is extensive histopathological, and *in vitro* and *in vivo* experimental evidence with various animal models of tissue fibrosis as well as correlational and descriptive studies from human patients implicating tissue remodeling/profibrotic (M2-like) monocytes or macrophages as the primary mediators of tissue fibrosis and numerous studies have explored their role in various human fibrotic disorders.

### Systemic Sclerosis and animal models of skin fibrosis

SSc is a systemic autoimmune disease characterized by progressive fibrosis of skin and multiple internal organs and severe microvascular alterations [191]. SSc pathogenesis is complex and poorly understood [147-149,192,193]. The severe fibrotic process results from ECM overproduction by activated fibroblasts triggered by complex paracrine and autocrine interactions between various cells including fibroblasts, endothelial cells and macrophages. Multiple reports have described the macrophage subpopulations present in SSc patients and in the skin of murine models of SSc. Soluble levels of CD163, a protein exclusively expressed in monocytes and macrophages [194] associated with M2 monocytes/macrophages, are elevated in SSc patient serum and these high CD163 levels correlate with SSc disease severity [195-198]. A detailed analysis of monocyte/macrophage subsets in skin samples and PBMC isolated from SSc patients showed a marked increase in cells displaying the tissue remodeling/profibrotic (M2) macrophage markers CD163 or CD204 [199]. The elevated soluble CD163 present in SSc patient plasma may be explained by the higher levels of TNF-α converting enzyme that mediates shedding of the CD163 ectodomain [200] in PBMC of patients with early SSc [201]. Levels of CCR2, a chemokine receptor crucial for M2 macrophage activation were elevated in macrophages and various other cells types in SSc patients with early diffuse disease but not in patients with late or limited SSc [202]. A recent analysis of global chemokine expression in SSc described high CCL19 and CXCL13 levels present in the skin of patients with diffuse SSc, whereas CCL18 levels were increased in the skin of patients with either limited or diffuse SSc [203]. Of interest were the observations that CCL19 expression was induced by TLR pathway activation *in vitro* and colocalized *in vivo* by immunofluorescence with CD163^+^ macrophages [203]. Several reports described evidence that circulating monocytes and macrophages in patients with SSc-associated lung fibrosis displayed elevated levels of markers of the tissue remodeling/profibrotic (M2) phenotype including CD163, CCL18 and IL10 [204] and a recent investigation described increased expression of multiple alternative macrophage activation markers that correlated with progression of SSc associated pulmonary fibrosis [205]. Although affected SSc tissues display perivascular accumulation of macrophages [150] the mechanisms responsible have not been elucidated, however, recent studies in two murine models of tissue fibrosis have implicated MMP12, a macrophage and endothelial cell secreted elastase [206,207].

Human and mouse skin also contain multiple and distinct subsets of DC [208-212] and in light of the important functions of DC in innate and adaptive inflammation, a role for these cells in the development of SSc has been postulated. Indeed, two reports have described decreased numbers of dendritic cells in skin isolated from human SSc patients compared with skin from normal donors [213,214] although this decrease occurred early in the progression of the disease and was present even in patients with a disease duration of 1 year of less and were substantially less abundant in patients with only mild fibrosis. An analysis of the response of human monocyte-derived DC isolated from SSc patients found an altered response to TLR stimulation in DC from patients with either the limited or diffuse form of SSc compared to those of normal patients with increased expression of IL6 and IFN-α and decreased expression of IL-12 whereas IL-10 expression was markedly increased only in SSc patients with diffuse disease [215]. SSc sera containing anti-topoisomerase autoantibodies was found to induce plasmacytoid DC differentiation in PBMC characterized by high type I IFN production [216]. Lastly, in the murine bleomycin model of skin fibrosis, CD11c^bright^ DC lose their even distribution in the skin and migrate to the dermis, in proximity to α-SMA^+^ myofibroblasts [217].

### Nephrogenic Systemic Fibrosis in mouse and human

NSF is a generalized fibrotic disorder occurring in some patients with chronic kidney disease exposed to Gd-based contrast agents (GdBCA) [218-220]. The clinical manifestations include severe and usually progressive skin induration, joint flexion contractures and fibrotic involvement of various internal organs. Recent studies have implicated remodeling/profibrotic (M2) macrophages in NSF pathogenesis. Human monocytes and tissue remodeling/profibrotic (M2-like) macrophages differentiated *ex vivo* by M-CSF and IL-10 increased the expression of the remodeling/profibrotic (M2)-associated cytokines IL-4, IL-13, VEGF, and numerous chemokines in an NF-κB-dependent manner following exposure to the GdBCA Omniscan. Furthermore, culture supernatants of the Gd-activated monocytes/remodeling/profibrotic (M2-like) macrophages induced α-SMA and types I and III collagen expression in cultured normal human dermal fibroblasts [181,182]. A related study showed that *ex vivo* differentiated remodeling/profibrotic (M2-like) macrophages were more responsive to GdBCA agents than inflammatory (M1-like) macrophages and this increased response was triggered by NLRP3 inflammasome activation [183].

### Idiopathic Pulmonary Fibrosis and pulmonary fibrosis in mouse animal models

IPF is a progressive interstitial lung disease of unknown etiology characterized by often progressive lung fibrosis causing irreversible loss of pulmonary function [221,222]. Several studies have shown that Ly6C^hi^ monocyte infiltration followed by differentiation into remodeling/profibrotic (M2-like) macrophages correlates with fibrosis progression and severity in bleomycin-induced pulmonary fibrosis [23,223-225]. Caveolin-1 (CAV1), the major protein component of caveolae and an important regulator of TGF-β signaling, is downregulated in the skin and lungs of SSc patients [226-229]. The profibrotic effects of CAV1 deficiency are likely related to impaired TGF-β receptor degradation, however, CAV1 downregulation in SSc interstitial lung disease has also been reported to increase tissue fibrosis by influencing monocyte migration and recruitment. This effect was mediated through induction of monocyte and fibrocyte expression of the remodeling/profibrotic (M2) macrophage marker CXCR4 that regulates monocyte retention in the bone marrow [230]. Furthermore, CAV1 and several other fibrosis associated cytokines and proteins, including IL-33, IL-9 and MMP28 regulate macrophage differentiation in pulmonary fibrosis in mice [231-236]. Alveolar macrophages in bronchoalveolar lavage fluid (BALF) in bleomycin-induced fibrosis expressed the remodeling/profibrotic (M2) factors IL-13 and TGF-β and had increased IL-33 expression whereas IL-33 knockout mice displayed impaired remodeling/profibrotic (M2) polarization and greatly diminished fibrosis [231]. Several recent studies have examined the role of MMP28 in the development and progression of pulmonary fibrosis, effects that appear to be mediated through the regulation of pulmonary macrophage phenotype [232,233]. Mice with a genetic deletion of MMP28 are protected from bleomycin-induced fibrosis and showed diminished remodeling/profibrotic (M2) responses [234]. Wild type MMP28 expression in mice is specific for Ly6C^hi^ monocytes that give rise to three subpopulations following M-CSF-mediated differentiation and activation by LPS or IL-4/IL-13. Inflammatory (M1-like) CD11b^low^ CD45^high^ cells lack MMP28 expression, whereas the remodeling/profibrotic (M2-like) CD11b^high^ CD45^high^ subpopulation and the CD11b^int^ CD45^int^ subpopulation display robust MMP28 expression [234]. Numerous cytokines and other macromolecules have been shown to participate in the molecular events leading to pulmonary fibrosis [235-238]. For example, IL-9 knockout mice had impaired remodeling/profibrotic (M2) macrophage polarization and were protected from silica-induced fibrosis [235], whereas, chronic IL-10 exposure in mice resulted in increased pulmonary fibrosis, increased remodeling/profibrotic (M2) macrophages in BALF and in the lungs as well as increased levels of CCR2 and CCL2 [236]. Conditional deletion of the tyrosine phosphatase Shp2 in mouse myeloid cells increased remodeling/profibrotic (M2) macrophage differentiation in response to IL-4 resulting in decreased susceptibility to pulmonary fibrosis [237]. Types I and III collagens caused increased CCL18 and IL-1ra production in human alveolar macrophages *in vitro,* as well as, increased expression of CCL2 and CD204, indicating that they had assumed a tissue remodeling/fibrotic (M2-like) phenotype [238].

In an evalution of the role of DC in IPF, lung biopsies isolated from human IPF patients, DC-SIGN^+^ DC were more numerous than in biopsies of normal donors and demonstrated a more perivascular localization, in some cases forming a novel type of organized lymphoid region within the lung [239,240]. In bleomycin induced pulmonary fibrosis in mice, mature Cd11c^+^ MHCII^+^ DC were found to accumulate in large numbers in proximity with T cells displaying an active or memory phenotype by day 7 following bleomycin exposure [241]. Treatment of mice with VAG539, an inhibitor of DC costimulatory molecules was shown to attenuate bleomycin-induced pulmonary fibrosis.

### Liver fibrosis in mice and humans

Liver fibrosis is one of the most frequent fibrotic disorders affecting humans and has multiple and diverse causes including hepatitis virus infections and excessive ethanol consumption, frequently resulting in liver failure and portal hypertension [242]. Increased tissue remodeling/fibrotic (M2) macrophage polarization has been described in hepatitis B virus and Schistosoma-induced hepatic fibrosis [243,244]. The number of monocytes in the liver is markedly increased following injury and liver monocyte subsets display distinct phenotypic characteristics [245,246]. The greatest proportion of monocytes in the normal and injured liver in humans belong to the CD14^++^CD16^+^ intermediate subset although this population dramatically increases following liver injury owing to increased transepithelial migration of this population relative to the classical CD14^++^CD16^-^ and the CD14^lo^CD16^+^ monocyte subsets as well as to their increased *in situ* differentiation. This intermediate population possesses high phagocytosis activity and secretes the inflammatory mediators TNF-α, IL-6, IL-8 and IL-1β as well as the tissue remodeling/fibrotic IL-13, CCL1, CCL2, CCL3, CCL5, CSF1 and GMCSF molecules. A central regulator of monocyte recruitment in response to CCL_4_ induced or non-alcoholic steatohepatitis in mice is the CCL2/CCR2 signaling pathway. Upon injury, CCL2/CCR2 signaling induce infiltration of Ly6C^+^ inflammatory monocytes into the liver resulting in the promotion of liver fibrosis as well as angiogenesis [247-250]. CCR2/CCR6 deficient mice fail to recruit Ly6C^+^ monocytes and are thus protected from CCL_4_-induced hepatic fibrosis [251,252]. Similarly, pharmacologic inhibition of CCL2 signaling diminishes macrophage infiltration and steatohepatitis [253] and actually promotes fibrosis resolution [254]. Significantly, mice lacking Tlr4, Tlr9 or MyD88, important upstream regulators of CCL2 expression, were similarly protected from steatohepatitis [251]. Conversely, the CX_3_CR1 fractalkine receptor acts to limit liver fibrosis by controlling the differentiation of monocytes and macrophages involved in fibrosis resolution [255]. In the absence of CX_3_CR1, intrahepatic monocyte differentiation skews to favor inflammatory TNF-α- and NO-producing macrophages that result in inflammation persistence and enhanced liver fibrosis. Taken together, these observations render the CCL2/CCR2 and the CX_3_CR1 pathways important targets for developing potential therapies for the treatment of liver fibrosis [19,256]. Several studies have described a unique population of macrophages with properties of both inflammatory (M1) and tissue remodeling/fibrotic (M2) macrophages, termed scar-associated macrophages, arising from recruited Ly6C^hi^ monocytes expressing high levels of MMP13 that mediate regression of liver fibrosis in animal models [86,131-136].

DC cells have also been proposed as regulators of liver fibrosis. Increased numbers of a mixed population of Cd11c^+^ DC have been observed in the livers of mice with hepatic fibrosis induced by TAA and leptin [257] with many of these cells also positive for MHCII and CD4. The isolated DC could stimulate NK and T cells both *in vitro* and *in vivo*. Cd11c^+^ cell depletion in these mice reduced the expression of TNF-α, IL-6 and other proinflammatory cytokines. Co-culture of Cd11c^+^ DC with hepatic stellate cells caused increased proinflammatory cytokine expression and increased stellate cell proliferation and it was shown that this effect was dependent on TNF-α [258].

### Renal and cardiac fibrosis in mice and humans

Numerous disorders cause kidney fibrosis including glomerulonephritis, diabetic nephropathy, SSc with kidney involvement and rare disorders such as immunoglobulin A nephropathy [158]. Although inflammation is an important component of renal fibrosis [158] the role of macrophage phenotype changes in the pathogenesis of these diseases has only just begun to be investigated. Injured kidneys selectively recruit Ly6C^hi^ monocytes and these recruited monocytes differentiate into functionally distinct macrophage populations [18]. Analysis of human pediatric and adult immunoglobulin A nephropathy renal biopsies in another report found significant CD163^+^ or CD204^+^ tissue remodeling/fibrotic (M2)-like macrophage infiltration in close proximity to activated myofibroblasts [259], an intriguing observation since *ex vivo* differentiated macrophages have been shown to induce myofibroblast phenotypic changes and α-SMA expression in cultured fibroblasts [260]. Recently, an analysis of renal allograft biopsies from kidney transplant patients isolated one year post-transplantation found that the majority of infiltrating macrophages were CD68^+^ CD204^+^ tissue remodeling/fibrotic (M2) macrophages characterized by increased expression of IFN-γ-responsive genes and that macrophage infiltration strongly correlated with the subsequent development of renal fibrosis [261]. A role of DC in renal fibrosis has recently been examined. In a mouse model of UUO-induced renal fibrosis, the numbers of both Cd11c^+^ F4/80^+^ and Cd11c^+^ F4/80^-^ increased 24 hours after injury with both subpopulations displaying increased MCH2 and CD68 positivity, however Cd11c^-^ DTR-specific depletion either at 1 day pre-UUO or 5 days post-UUO had no effect on levels of type III collagen, α-SMA, TGF-β or TNF-α [262].

Cardiac fibrosis resulting from myocardial infarction or in association with other fibrotic disorders such as SSc or NSF involves accumulation of ECM proteins in the myocardium resulting in myocardial failure [263]. There has been recent interest in the role of macrophage polarization in the pathogenesis of cardiac fibrosis. It has recently been shown that inhibition of TLR2 decreased angiotensin II-induced cardiac fibrosis through a potent reduction in the tissue infiltration by inflammatory cells, particularly macrophages [264], and another study found that MMP28 regulated the fibrotic response to myocardial infarction in mice, with MMP28 deletion exacerbating cardiac fibrosis, an effect mediated by inhibition of M2 macrophage activation [265]. The role of CAV1 in cardiac interstitial fibrosis was examined in experimentally induced myocardial infarction in mice and reported that deletion of CAV1 resulted in increased numbers of tissue remodeling/fibrotic (M2) macrophages in the infarct zone and that infusion of the CAV1 scaffolding domain peptide abrogated the increased tissue remodeling/fibrotic (M2) macrophage accumulation and reduced cardiac fibrosis [266].

### Skeletal muscle fibrosis in mice and humans

As in other tissues, fibrosis can occur following injury to the muscle, or as a consequence of the normal aging process, or in association with a pathologic condition such as Duchenne muscular dystrophy [267]. Macrophage subpopulations perform opposing activities in muscle regeneration. Inflammatory (M1) macrophages secrete TNF-α and IL-1β mediating monocyte recruitment and removal of necrotic cells at early stages after the initiating event. Tissue remodeling/fibrotic (M2) macrophages secrete IL-10 and TGF-β and become more abundant at advanced stages of the process, facilitating tissue repair and healing and inactivating the initial inflammatory macrophages [268]. It has recently been shown in mice that a mutation in the C/EBPβ promoter impairs differentiation of tissue remodeling/fibrotic (M2) macrophages resulting in the ability to remove necrotic tissue but with defects in myofiber regeneration, likely caused by changes in arginine metabolism [269]. Shifts in macrophage phenotypes and competition for arginine can affect the severity of the muscle pathology seen in the mdx mouse model of muscular dystrophy by promoting cardiac and muscle fibrosis [270,271]. The expression of integrin-β3 in macrophages has been found to affect macrophage polarization and infiltration since mice lacking integrin-β3 show increased infiltration of tissue remodeling/fibrotic (M2) macrophages into injured muscle and that these infiltrating macrophages express higher levels of TGF-β1, increased TGF-β1 Smad signaling, impaired muscle regeneration and increased fibrosis [272].

Interestingly, diaphragms from mdx mice display markedly increased expression of CCR2 and its chemokine ligands and increased recruitment of Ly6C^hi^ monocytes that differentiate into CD11b^hi^ macrophages [273]. Knockout of CCR2 abrogates Ly6C^hi^ monocyte recruitment and prevents skewing of macrophage differentiation to favor the inflammatory (M1) phenotype and decreases muscle fibrosis. The PDE5 inhibitor sildenafil significantly reduced muscle weakness after 14 weeks of treatment in mdx mice and slowed the establishment of mdx diaphragm fibrosis accompanied by a reduction in MMP13 expression and normalized expression levels of TNF-α [274]. The proposed antifibrotic agent imatinib mesylate markedly reduced limb muscle necrosis, inflammation and fibrosis accompanied by inhibition of c-able and PDGFR phosphorylation and the suppression of TNF-α and IL-1β expression in mdx mice [275,276].

### Opportunities for utilizing macrophage polarization as a clinical and therapeutic tool

Elucidation of the role of monocyte/macrophage differentiation and polarization in the context of fibrosis pathogenesis and resolution will provide novel opportunities for designing therapies to restore the balance between normal tissue repair and pathologic fibrosis [277]. Several new strategies are being explored as therapeutic modalities to modulate the crucial contribution of monocytes/macrophages to fibrotic disease pathogenesis. Monocyte recruitment is being targeted based on the differential expression of chemokine receptors by monocyte/macrophage subpopulations to encourage migration and tissue accumulation of beneficial macrophages or to prevent the recruitment of harmful profibrotic macrophages. Since CCR1 and CCR2 are more highly expressed on Ly6C^hi^ inflammatory (M1)-like monocytes whereas CCR5 and CX3CR1 are more characteristic of the Ly6C^low^ tissue remodeling/fibrotic (M2)-like monocytes [11-13], targeting CCR2 and CCR6 signaling would be expected to interfere with Ly6C^hi^ monocyte recruitment [278,279]. Although this approach is promising its success is uncertain since the Ly6C^hi^ population can differentiate into the Ly6C^low^subpopulation *in situ* following migration into the affected tissues.

Autologous monocyte/macrophage transfer can also be employed to alter monocyte subpopulations. A recent analysis utilizing such an approach showed that mesenchymal stromal cell transfer following coronary artery occlusion-induced myocardial infarction increased the proportion of tissue remodeling/fibrotic (M2) macrophages at the infarct site compared with monocyte transfer [280] accompanied by increased macrophage cytokine secretion, and improved infarct healing and repair. The beneficial effects were mediated by macrophages as they were abrogated by transient macrophage depletion [280]. This approach could introduce an enriched potentially therapeutic subpopulation of autologous cells or of cells genetically modified to target specific macrophage pathways to ameliorate fibrosis induction or to induce fibrosis resolution. However, one potential drawback of this approach is that the phenotype of the transferred monocytes/macrophages could be reprogrammed *in situ* by the microenvironment of the destination organ [281,282].

Antibody-conjugated microparticles can specifically target macrophage subpopulations based on differential properties of the nanoparticles used [283] or by conjugation with subpopulation specific markers [284,285]. Such an approach could be adapted to deliver phenotype modifying or cell lethal contents to specific macrophage populations. For example, administration of a peptide sequence unique to tissue remodeling/fibrotic (M2) macrophages identified by subtractive phage biopanning and fused to the KLA proapoptotic peptide [286] decreased mortality in CT-26 tumor-bearing mice by inducing a decreased number of tumor-associated macrophages [287]. These approaches alone, or in combination, offer the potential to nullify the fibrotic potential or to enhance the fibrosis resolving properties of specific macrophage subpopulations.

## Conclusions

Although monocyte/macrophage polarization in tissue fibrosis pathogenesis and resolution is an area of intense interest, the actual role of this phenomenon *in vivo* remains unclear. Extensive *in vitro* experimentation implicates M2 macrophages as the primary mediators of tissue fibrosis, however, the data from *in vivo* studies present a more complex picture. The M1 macrophage subpopulation is considered as the primary mediator of inflammation whereas the M2 macrophage subpopulation is considered to be the primary mediator of wound healing and pathologic fibrosis. The role of macrophage phenotypes *in vivo*, however, may be less definitive owing to the plasticity of monocyte/macrophage subpopulation phenotypes in response to local environmental factors. Numerous studies of organ-specific monocyte/macrophage subpopulations in specific organs often suggest that tissue remodeling/fibrotic (M2)-like monocytes and macrophages mediate fibrosis, however, other studies indicate a role for inflammatory (M1)-like macrophages or a requirement for both major subpopulations in the development of tissue fibrosis and in the pathogenesis of fibrotic disorders. Increasing knowledge about the differential properties of the multiple monocyte and macrophage subpopulations will undoubtedly lead to novel therapeutic strategies for the selective targeting of specific subpopulations involved in the development of pathologic fibrotic conditions. Among these novel approaches several have already shown their feasibility including for example, modifying monocyte recruitment and macrophage differentiation, transfer of autologous monocyte/macrophage populations, and the use of nanoparticles and lysosomes designed to be selectively bound by specific subpopulations and to modify their functional capabilities or induce their selective elimination.

## Abbreviations

AnxA1: Annexin A1
Arg1: Arginase 1
α-SMA: Alpha smooth muscle actin
CCL: C-C chemokine ligand
CCR: C-C chemokine receptor
CD: Cluster of Differentiation
Chi3l3: Chitinase 3-like 3
CLRs: C-type lectin receptors
CSF-1: Colony stimulating factor-1
CXCR: C-X-C chemokine receptor
CX3CR: C-X3-C chemokine receptor
DAMPs: Damage associated molecular patterns
ECM: Extracellular matrix
EndoMT: Endothelial-to-mesenchymal transition
GM-CSF: Granulocyte-macrophage-colony stimulating factor
IFN: Interferon
IGF-1: Insulin-like growth factor 1
IκBα: Nuclear factor of kappa light polypeptide gene enhancer in B cells inhibitor, alpha
IL: Interleukin
iNOS: Inducible nitric oxide synthase
IPF: Idiopathic pulmonary fibrosis
IRF: Interferon regulatory factor
ISG20: Interferon stimulated gene 20
LPS: Lipopolysaccharide
Ly6C: Lymphocyte antigen 6C
M-CSF: Macrophage-colony stimulating factor
MHCII: Major histocompatibility complex II
MMP: Matrix metalloproteinase
MyD88: Myeloid differentiation primary response gene 88
NF-κB: Nuclear factor kappa B
NLRs: NOD-like receptors
NLRP3: NLR family, pyrin domain-containing protein 3
NSF: Nephrogenic systemic fibrosis
PAMPs: Pathogen associated molecular patterns
PBMC: Peripheral blood mononuclear cell
ROS: Reactive oxygen species
SSc: Systemic sclerosis/scleroderma
STAT: Signal transducer and activator of transcription
TGF-β: Transforming growth factor, beta
Th1: T-helper 1
Th2: T-helper 2
TIMPs: Tissue inhibitors of metalloproteinases
TNF-α: Tumor necrosis factor alpha
TLR: Toll-like receptor
VEGF: Vascular endothelial growth factor
